# Tobacco Advertisements And Promotion Industry On Smoking In Tanzania: A Review Of Negative Public Health Implications For Current And Future Generations

**DOI:** 10.1186/1617-9625-3-2-41

**Published:** 2006-08-15

**Authors:** Stephen ED Nsimba, Steve Sussman

**Affiliations:** 1 Muhimbili University College of Health Sciences (MUCHS), Dept of Clinical Pharmacology, Dar-es-Salaam-Tanzania-East Africa; 2 Johns Hopkins University, Bloomberg School of Public Health, Baltimore, MD, USA; 3 Departments of Preventive Medicine and Psychology and Institute for Health Promotion and Disease Prevention Research, Alhambra, CA, USA

## Abstract

**Summary:**

Smoking places a burden on the health care system in developed countries but even more so in the already compromised health services in developing countries. The promotion of tobacco is a root cause of continued consumption even after highlighting the effects. The main aim of this news analysis is to discuss various strategies used by the tobacco industry in promoting tobacco use in Tanzania.

## Background

So far in Tanzania, there are no national tobacco programs targeting urban and rural communities. The only warnings available are on cigarette packs which say: "cigarette smoking is dangerous to your health," "smoking is not good for your health" or "smoking is dangerous for your life". There are several cigarette brands available in the country such as: Sweet Menthol, Sportsman, Embassy, Club, Baridi, Safari and Nyota. For example, as currently aired on TV and radio, the Sweet Menthol cigarette brand is being advertised and heavily promoted as "being very good or excellent for use". The cover of the cigarette package provides an image of cool flavor, which may be enjoyed in a warm climate. Sportsman provides an image of a horse (a Western cowboy image, manliness) and Embassy provides an image of crest (a Western royalty-like image, success image). These promotions might serve to counteract against the warning label information provided, which composes a very small portion of the cigarette pack, though no empirical research is being completed to assess that possibility.

In addition to radio and TV ads, there are many tobacco industry-sponsored billboard advertisements which promote cigarette smoking while also giving warnings against cigarette smoking. As on the cigarette packs these warnings take up a relatively small proportion of the labeled surface area.

## Trade and Current Regulations

Because of free trade liberalization, there are transnational tobacco companies aggressively marketing cigarettes, like the DIMON Incorporated from the US http://www.dimon.com/about/wo.htm, which is the world's second largest independent leaf tobacco professor and merchant and which even provides money to help local tobacco growers continue to produce tobacco. These companies are increasingly encouraging more tobacco growing and are also targeting youths, adolescents and women (Jacobson et al, 1996). They engage in numerous promotions that are usually accompanied with advertisements such as "smoking is socially desirable" and they relate smoking to manliness and success. This is likely to win most of the young generation. With the effect of globalization and the easing of some social-cultural-norms and values, most of the general population in the country is at risk of increasing tobacco smoking use [1].

Advertisements and other promotional activities on cigarettes gives adolescents reassurance especially for highly advertised brands and, thus, have both predisposing as well as reinforcing effects. Furthermore, there is good evidence that smoking has symbolic appeal for many young people. Young smokers tend to be perceived as relatively "tough" [2,3].

The situation in Tanzania is worrisome as far as tobacco control strategies are concerned. The government (Ministry of Health) has imposed some very partial or passive bans on the tobacco industry such as making them put warning signs on each cigarette pack. However, some senior elites smoke in their offices (governmental or non-governmental institutions) and if one visits such offices one may think that the Tanzania Cigarette Company Plant has moved to that particular office. Generally, ashtrays are usually on the tables. People still smoke openly in public places such as dance halls or in classrooms for most primary and secondary school teachers. There is need to re-educate and remind these people and at the same time impose strict rules not to smoke in offices or in class rooms.

Studies have shown that in tobacco growing countries in Africa, farmers are happy and willing to increase the output [7,8]. Tobacco farmers in Africa are reluctant to substitute tobacco with alternative crops that would attract price incentives or production support.

## Need for Restrictions

Strong tobacco control measures need to be instituted by the Tanzanian government by involving the Ministries of Health, Agriculture, Finance, Economic and Planning, Finance, National Education and Higher Education, and Science and Technology before tobacco related diseases reach an alarming situation in the country. Tobacco preventive or counter-promotion activities should target both the tobacco industry and the most vulnerable groups [4-6,9]. For example, Sussman and colleagues (under review) reported four phases of tobacco control that may lead to a mature stance against tobacco promotions. As tobacco control policies mature, they pass from a stage where a country only consider the possibility that tobacco use may be a problem and assessment begins, to where initial policy and education efforts begin, to where major policy changes are enacted and enforced, and finally to where transnational tobacco industry reactions are anticipated and responded to. Tanzania will need to go through the latter phases to mature into a strong tobacco control stance.

Hopefully, Tanzania will one day ratify key provisions in the Framework Convention on Tobacco Control (FCTC) treaty, a world-wide collaborative action that encourages countries to (a) enact comprehensive bans on tobacco advertising, promotion and sponsorship; (b) obligate the placement of rotating health warnings on tobacco packaging that cover at least 30 percent (but ideally 50 percent or more) of the principal display areas and can include pictures or pictograms; (c) ban the use of misleading and deceptive terms such as "light" and "mild"; (d) protect citizens from exposure to tobacco smoke in workplaces, public transport and indoor public places; (e) combat smuggling, including the placing of final destination markings on packs; and (f) increase tobacco taxes (see http://www.FCTC.org). The FCTC also contains numerous other measures designed to promote and protect public health, such as mandating the disclosure of ingredients in tobacco products, providing treatment for tobacco addiction, encouraging legal action against the tobacco industry, and promoting research and the exchange of information among countries.

From the public health point of view – "prevention is better than cure". It should therefore be born in mind by the government that it is much more expensive to treat diseases caused by tobacco smoking when they occur than to prevent tobacco smoking incidence. Interventions should be directed towards counteracting these industrial advertisements and promotional tobacco activities targeted to the audience in the country in order to rescue the youth at risk. Certainly, government increases in taxation and public area no-smoking policies should be attempted in the near-future.

## Competing interests

The authors declare that they have no competing interests.
